# Proteomic profiling of antibody-inducing immunogens in tumor tissue identifies PSMA1, LAP3, ANXA3, and maspin as colon cancer markers

**DOI:** 10.18632/oncotarget.23583

**Published:** 2017-12-22

**Authors:** Qian Yang, Michael H. Roehrl, Julia Y. Wang

**Affiliations:** ^1^ Department of Laboratory Medicine and Pathobiology, University of Toronto, Toronto, Canada; ^2^ Ontario Cancer Institute, University Health Network, Toronto, Canada; ^3^ Department of Pathology, Memorial Sloan Kettering Cancer Center, New York, NY, USA; ^4^ Curandis, Boston, MA, USA

**Keywords:** colon cancer, antibody, immunogen, precision diagnostics, immunotherapy

## Abstract

We hypothesized that cancer tissue immunogens – antigens capable of inducing specific antibody production in patients – are promising targets for development of precision diagnostics and humoral immunotherapies. We developed an innovative immuno-proteomic strategy and identified new immunogenic markers of colon cancer.

Proteins from cancers and matched normal tissues were separated by 2D gel electrophoresis and blotted with serum antibodies from the same patients. Antibody-reactive proteins were sequenced by mass spectrometry and validated by Western blotting and immunohistochemistry.

170 serum antibody-reactive proteins were identified only in cancerous but not matched normal. Among these, proteasome subunit alpha type 1 (PSA1), leucine aminopeptidase 3 (LAP3), annexin A3 (ANXA3), and maspin (serpin B5) were reproducibly found in tissues from three patients. Differential expression patterns were confirmed in samples from eight patients with various stages of colon adenocarcinoma and liver metastases.

These tumor-resident proteins and/or their associated serum antibodies may be promising markers for colon cancer screening and early diagnosis. Furthermore, tumor tissue-specific antibodies could potentially be exploited as immunotherapeutic targets against cancer. More generally, proteomic profiling of antibody-inducing cancer-associated immunogens represents a powerful generic method for uncovering the tumor antigen-ome, i.e., the totality of immunogenic tumor-associated proteins.

## BACKGROUND

Colon cancer is amongst the most common cancers and poses a major health burden worldwide [1]. Every year more than one million new cases of colon cancer are diagnosed and half a million deaths by colon cancer are reported [2]. When diagnosed early, localized colon cancer can be surgically removed, and the 5-year survival rate of patients can be as high as over 90%. However, patients with later stages of colon cancer and metastases have an average survival rate of less than 10% [3]. Development of effective markers for early diagnosis, prognosis, and therapeutic decision-making is therefore essential for advancing of colon cancer management [4].

Cancer development is a complex multistep process that is influenced not just by genetic changes but, more importantly, by alterations of downstream molecular expression and function [5]. Cancer develops through steps of initiation, promotion, progression, and metastasis. The initial step is commonly attributed to genetic changes that may be spontaneous or triggered by carcinogens. Cells gradually become malignant through a progressive series of alternations such that they lose their normally regulated functional abilities and undergo uncontrolled growth and proliferation [6, 7]. Eventually, cancer cells invade nearby tissues or migrate to distant tissue via circulatory systems, leading to metastasis.

Cancer development is accompanied by changes in the molecular makeup of the cancer cells and their surrounding stroma. In particular, proteins may exhibit a range of variations in expression quantities, post-translational modifications, folding, biologic half-lives, or degradation pathways. In early stages of cancer evolution, the immune surveillance system has the ability to recognize nascent cancer cells and to develop cellular or humoral immunological responses. Antibodies against a number of tumor-associated antigens have been detected in early stages of various types of cancer [8–12]. For example, anti-p53 and anti-p16 were detected in 10-20% of patients with various types of cancer [9, 12]. Anti-p62 was positive in 21% of patients with liver cancer [13]. An autoantibody response to p90/CIP2A was detected in breast cancer [14]. These antibodies are generally absent in healthy individuals, but are persistent and stable in sera of cancer patients.

As cancer progresses, tumor cell variants that are poorly immunogenic or even immunosuppressive prevail, which can elude the immune system and evade recognition and destruction by the immune defense. Consequently, these cells can survive and grow within an immune-competent environment. Moreover, cancer cells can actively suppress immune responses by different pathways [6, 15]. Hence, when designing effective immunotherapies, it is crucial to distinguish cancer-associated molecules that are able (immunogenic) vs. unable (non-immunogenic) to evoke immune responses.

In this study, we focused on discovering overexpressed immunogenic proteins in colon cancer tissue that are able to induce circulating antibody production in patients. We developed a serum antibody screening method to identify these molecules. We used the cancer patients’ own sera as the primary antibody source to screen for differential protein reactivity in autologous matched normal and cancerous tissue pairs. We identified and then validated a number of immunogenic proteins as potential markers for colon cancer.

## RESULTS

### Differential protein expression profiles in cancer and normal tissue

**Figure 1:**
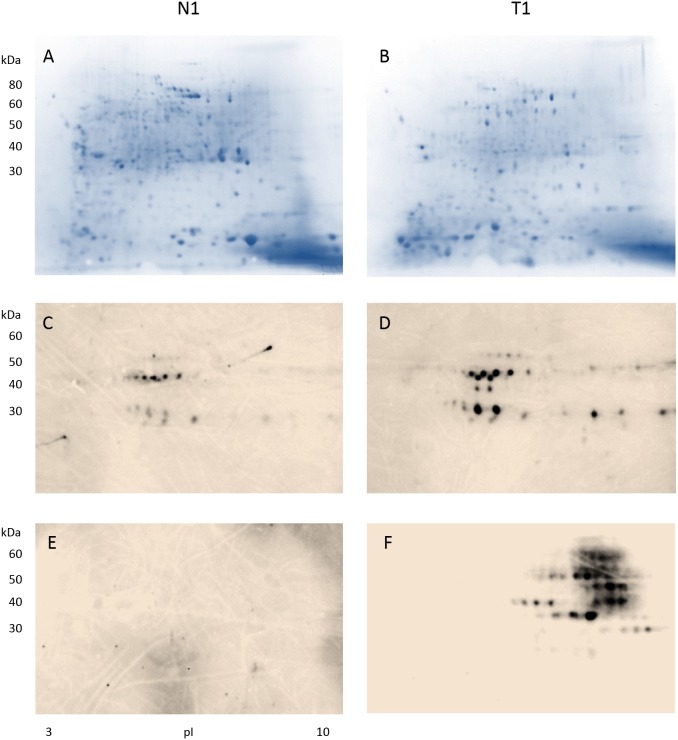
Detection of antibody-reactive proteins in colon tissue extracts from a male patient with stage II colon adenocarcinoma Proteins extracted from the normal colon tissue (CN1) and the tumor colon (CA1) portions were separated in 2D gels **(A-B)**, blotted with a control serum containing high-titer autoantibodies to reveal cancer non-specific autoantigens found in autoimmune diseases **(C-D)**, and blotted with serum from the cancer patient to uncover cancer-specific immunogens that induced antibodies in the serum **(E-F)**. Note that only proteins extracted from the tumor tissue **(F)** showed reactivity with antibodies in the patient serum.

**Figure 2:**
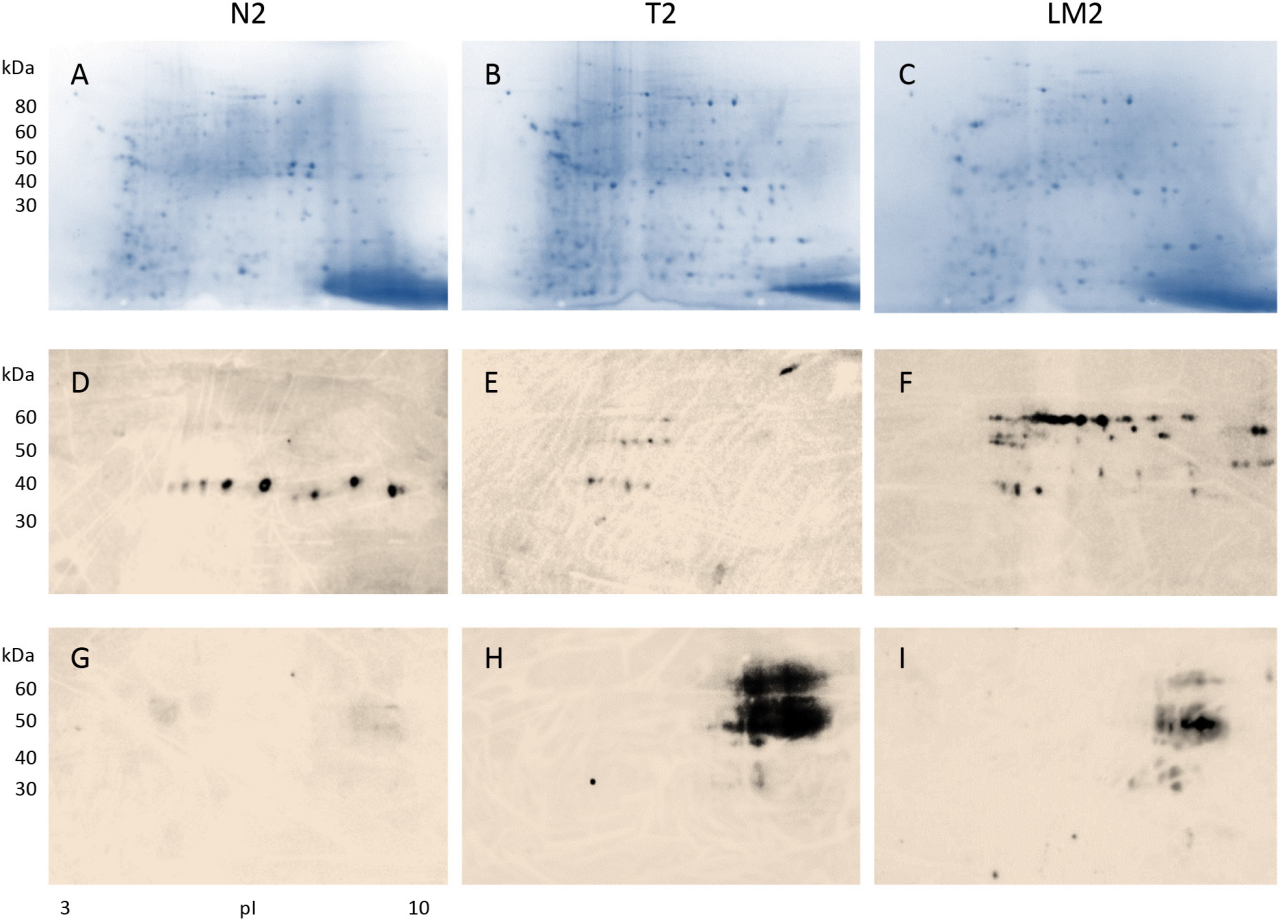
Detection of antibody-reactive proteins in tissue extracts from a female patient with stage IV colon cancer and liver metastasis Proteins extracted from tissue of normal colon (**A**: CN2) and colon adenocarcinoma (**B**: CA2), and liver metastases (**C**: LM2) were analyzed by 2D electrophoresis. Protein extracts were blotted with a control serum containing high-titer autoantibodies from an autoimmune disease patient to exclude autoantigens non-specific to the cancer patient **(D-F)**. Cancer-specific proteins that induced serum antibodies were revealed by blotting with serum from the cancer patient **(G-I)**. Note that cancer-specific immunogen-antibody reactivity patterns in colon tumor (H) and liver metastatic tissue (I) appear similar.

Matched pairs of normal and cancerous colon tissues were obtained from patients undergoing surgical colectomy procedures. For patients whose colon cancer had metastasized to the liver, tissue samples from the liver metastases were also obtained. The tissue protein extraction procedure was optimized, and proteins were extracted from all matched tissue pairs or sets in parallel with the optimized protocol. Extracted proteins were initially assessed by 1D SDS PAGE and then separated and analyzed by 2D gel electrophoresis. From proteins extracted from each tissue sample, triplicates of high quality 2D gels were prepared: one gel was used for sequencing of differentially expressed proteins by mass spectrometry (Figures 1-2, top row), another for detection of reactivities with standard autoantibody control serum by 2D Western blotting (Figures 1-2, middle row), and a third for discovery of reactivities with the cancer patient’s own serum (Figures 1-2, bottom row).

From each tissue extract, more than 500 distinct protein spots were typically detectable on 2D gels (Figures 1A-1B, 2A-2C). By computational gel image comparison, on average about 80 protein spots were found to be differentially expressed between normal and tumor tissue, when spots exhibiting at least 2-fold intensity changes were considered. When sequenced by mass spectrometry, each spot on the 2D protein gels often yielded tens to hundreds of protein identities. Therefore, the number of protein identities is enormous if no further selection criterion is applied. We then decided to narrow our focus to proteins that are immunogenic in cancer tissue, i.e., proteins in cancer tissue that are capable of inducing the production of specific serum antibodies in the patient.

### Cancer-specific antibodies and protein immunogens

In order to identify cancer-specific immunogen markers, proteins extracted from the tumor tissue and its matching normal tissue were interrogated with the patient’s own serum by 2D Western blotting (Figures 1-2, bottom row). To further distinguish tumor-specific immunogens vs. tumor non-specific autoantigens that react with common or naturally occurring autoantibodies, the proteins from tissue extracts were also probed with control sera containing high titers of autoantibodies from unrelated non-oncologic autoimmune disease patients (Figures 1-2, middle row).

For the initial discovery stage, specimens from 2 patients, a male with stage II (pT3 pN0) colon adenocarcinoma (Figure 1) and a female with stage IV (pT3 pN2b pM1a) colon adenocarcinoma and liver metastases (Figure 2), were examined in depth. We purposely chose two patients who differed with regard to gender and cancer stage hypothesizing that common markers that were shared between their tumors might be applicable to a wider spectrum of colon adenocarcinoma cases. Shared markers identified from these two patients were further examined in a larger cohort of patients as presented later in the paper.

Results from the stage II male colon cancer patient are shown in Figure 1. When blotted with control autoantibodies, 15 reactive spots appeared against proteins from normal colonic mucosa (Figure 1C) whereas 27 reactive spots appeared against proteins from cancerous tissue (Figure 1D). Proteins extracted from cancer reacted more strongly with the control autoantibodies. However, overall autoantibody-reactive blotting patterns from proteins extracted from normal vs. cancer tissue appear to be similar (Figures 1C vs. 1D), suggesting that autoimmune autoantigens present in normal and cancerous tissue are similar or, in other words, not cancer-specific.

In contrast, when protein extracts were blotted with the cancer patient’s own serum, the antibody-reactive profiles were drastically different (Figure 1E-1F). There were about 8 horizontal molecular weight zones comprising 32 distinct spots that reacted strongly with antibodies in the patient’s serum (Figure 1F). Strikingly, no reaction was detected between serum antibodies and benign mucosal proteins (Figure 1E). Furthermore, comparing the blots of Figure 1D vs. 1F, it is evident that cancer-specific immunogens in colon cancer tissue (Figure 1F) are significantly different from autoantigens of autoimmunity (Figure 1D). Therefore, the patient produced serum antibodies that are specifically directed against certain proteins in his colon cancer. The findings have three implications: (i) the cancer patient developed cancer-specific antibodies that are different from commonly occurring autoantibodies, (ii) the cancer-specific antibodies recognize a small number of proteins expressed by the cancer tissue only, (iii) these antibody-reactive proteins are either absent or different in normal tissue.

### Presence of colon cancer-specific immunogens in liver metastases

Results from a female patient with stage IV colon cancer and liver metastases are shown in Figure 2. Proteins were extracted from normal and cancerous colon tissues as well as the metastatic cancer tissue and separated in 2D gels. After blotting with control serum that contained high titers of autoantibodies from an autoimmune disease patient, 1 horizontal zone of about 10 reactive spots was detected from benign colonic mucosal proteins (Figure 2D), while 3 horizontal zones of reactive spots were detected from colon cancer tissue proteins (Figure 2E). The cancerous tissue metastatic to the liver yielded about 36 spots reactive with autoimmune control serum (Figure 2F). Comparison of Figures 1C-1D and 2D-2F shows that, although there is some resemblance, autoantigen patterns in the two patients are largely different and not cancer-specific.

In contrast, when proteins from normal and cancer tissue were blotted with the colon cancer patient’s own serum, strong reactions were detected against a number of proteins from the primary colon cancer and cancer metastatic to the liver (Figure 2H-2I), while no reaction was detectable against proteins from normal colonic mucosa (Figure 2G). These observations are similar to the findings from the stage II male colon cancer patient above. Strikingly, the reactivity patterns of primary cancer and metastasis resemble each other, suggesting that shared cancer-specific immunogens are expressed in both the primary and the metastasis. Thus metastatic tumor cells appear to maintain the same or similar immunogenic protein makeup compared to the primary.

The similarity of cancer-immunoreactive signatures (Figures 1F, 2H, 2I) from our 2 patients with different stages of colon adenocarcinoma suggested that there were at least some protein reactivities that were shared between both patients. The identification of these shared immunogens was our next focus.

### Protein identification by mass spectrometry

Protein spots reacting with cancer serum antibodies were cut from 2D gels, digested with trypsin, and sequenced by mass spectrometry (MS). Protein identities were accepted only if more than two peptides in the sequence were confirmed. Proteins identified from the cancer tissue were compared with those from the matched normal mucosa. After proteins that were found in both the normal and cancer tissue were eliminated, 170 proteins were found in cancer tissues only (Table 1).

**Table 1:** Proteins identified in tumor tissue but not in normal tissue

Protein name	Accession number	MW (kDa)	Peptides identified	Protein coverage	Tissue origin^*^
**Serpin B5 (Maspin)**	**SPB5_HUMAN**	**42**	**18**	**47%**	**1/2/2L**
**Leucine aminopeptidase 3 (LAP3)**	**AMPL_HUMAN**	**53**	**11**	**27%**	**1/2/2L**
**Annexin A3 (ANXA3)**	**ANXA3_HUMAN**	**36**	**9**	**35%**	**1/2/2L**
**Proteasome subunit alpha 1 (PSMA1)**	**PSA1_HUMAN**	**30**	**9**	**34%**	**1/2/2L**
**Septin-11**	**D6RER5_HUMAN**	**50**	**14**	**26%**	**1/2/2L**
Isoform 2 of Annexin A2	ANXA2_HUMAN	40	22	46%	1
Actin-related protein 2	ARP2_HUMAN	45	6	19%	1
Annexin A1	ANXA1_HUMAN	39	5	19%	1
Malate dehydrogenase, mitochondrial	MDHM_HUMAN	36	5	19%	1/2
Gamma-glutamyl byfrolase	GGH_HUMAN	36	5	15%	1
Glutaredoxin-3	GLRX3_HUMAN	37	4	17%	1
HLA class I histocompatibility antigen, A-43 alpha chain	IA43_HUMAN	41	4	18%	1
Cathepsin B	CATB_HUMAN	38	3	14%	1
EGF containing fibulin-like extracellular matrix protein	B4DW75_HUMAN	39	3	10%	1
F-actin-capping protein subunit alpha-1	CAZA1_HUMAN	33	6	20%	1/2L
Pyridoxal kinase	F2Z2Y4_HUMAN	31	6	22%	1
AH receptor-interacting protein	A1P_HUMAN	38	6	22%	1
Guanine nucleotide-binding protein G(I)/G(S)/G(T) subunit beta-1	GBB1_HUMAN	37	4	14%	1
Voltage-dependent anion-selective channel protein 1	VDAC1_HUMAN	31	3	13%	1/2
Isoform 2 of ficolin-3	FCN3_HUMAN	32	3	10%	1
Gamma-soluble NSF attachment protein	SNAG_HUMAN	35	3	13%	1
Isoform 3 of filamin-binding LIM protein 1	FBLI_HUMAN	31	3	11%	1
Glutathione S-transferase P	GSTP1_HUMAN	23	6	36%	1
Sp/P20160/CAP7_HUMAN Azurocidin	CAP7_HUMAN	27	4	14%	1
Ras-related protein Rab-11A	RB11A_HUMAN	24	3	15%	1
Peptidoglycan Recognition protein 1	PGRP1_HUMAN	22	5	32%	1
Complement factor D	CFAD_HUMAN	27	4	20%	1
Alpha-enolase	ENOA_HUMAN	47	36	42%	1/2
Protein disulfide isomerase family A, membrane 3, isoform CRA_bne	G5EA52_HUMAN	55	27	38%	1
Isoform 2 of heat shock cognate 71kD protein	HSP7C_HUMAN	54	14	28%	1
Procollagen c-endopeptidase enhancer 1	PCOC1_HUMAN	48	10	27%	1
Septin 6	B1AMS2_HUMAN	49	9	20%	1
Rab GDP dissociation inhibitor beta	E7EU23_HUMAN	51	7	18%	1
Haptoglobin	HPT_HUMAN	45	4	12%	1
Coronin	A7MAPO_HUMAN	54	3	7%	1
Phosphoglycerate kinase 1	PGK1_HUMAN	45	3	11%	1
Isoform 3 of chitotriosidase-1	CHITI_HUMAN	49	3	8%	1
Isoform 3 of L-lactate dehydrogenase A chain	LDHA_HUMAN	40	4	11%	1
Enlongation factor 1-alpha 1	EF1A1_HUMAN	50	4	9%	1
Acid ceramidase	E7EMM4_HUMAN	42	4	11%	1
T-complex protein 1 subunit alpha	TCPA_HUMAN	60	10	28%	2
Succinyl-CoA-Ketoacid coenzyme A transferase 1, mitochondrial	SCOT1_HUMAN	56	13	38%	2
DnaJ homolog subfamily C member 3	DnJC3_HUMAN	58	13	35%	2
NOL1/NOP2/Sun domain family, member 2, isoform CRA_b	G3V1R4_HUMAN	59	5	15%	2
UDP-N-acetylhexosamine pyrophosphorylase-like protein	UAP1L_HUMAN	57	5	13%	2
Serine/threonine-protein kinase PAK2	PAK2_HUMAN	58	7	18%	2
Zyxin	B4DQR8_HUMAN	52	3	11%	2
Golgi-associated PD2 and coiled-coil motif-contain protein	F5H1Y4_HUMA	51	3	6%	2
Coronin-1B	COR1B_HUMAN	54	3	6%	2
Annexin, AII	ANXII_HUMA	54	4	29%	2
Thioredoxin reductase 1, cytoplasmic	B2R5P6_HUMAN	55	7	17%	2
E3 ubiquitin-protein ligase XIAP	XIAP_HUMAN	57	8	23%	2
Tripartite motif-containing protein 65	TRI65_HUMAN	57	5	21%	2
Nucleoprin p54	B4DT35_HUMAN	51	15	42%	2
D-3-phosphoglycerate dehydrogenase	SERA_HUMAN	57	12	30%	2
Isoform 2 of Alpha-amino adipic semialdehyde dehydrogenase	AL7A1_HUMAN	55	7	22%	2
Isoform 2 of Endophilin-B1	SHLB1_HUMAN	43	5	14%	2
Vasodilator-stimulated phosphoprotein	VASP_HUMAN	40	5	17%	2
Endophilin-A2	SH3G1_HUMAN	41	3	10%	2
Uroporphyrinogen decarboxylase	DCUP_HUMAN	41	3	10%	2
Short/branded chain specific acyl-CoA dehydrogenase, mitochondria	ACDSB_HUMAN	47	3	9%	2
UPF0160 protein MYG1, mitochondrial	MYG1_HUMAN	42	3	12%	2
Zinc finger cccH domain-containing protein	ZC3HF_HUMAN	49	6	18%	2
Synaptosomal-associated protein 29	SNP29_HUMAN	29	4	22%	2
N-myc-interactor	NM1_HUMAN	35	5	23%	2
HCG 2043421, isoform CRA-C	134E3T4_HUMAN	25	5	23%	2
Interferon-induced 35 kD protein	IN35_HUMAN	32	3	21%	2
Chloride intracellular channel protein 3	CLIC3_HUMAN	27	3	13%	2
Putative WAS protein family homolog 4 (fragment)	H3BV49_HUMAN	33	3	12%	2
DNA-directed RNA polymerase I, II, and III subunit	RPAB1_HUMAN	25	7	41%	2
Putative ATP-dependent Clp protease protedytic subunit, mitchondrial	CLPP_HUMAN	30	5	27%	2
Cathepsin G	CATG_HUMAN	29	4	16%	2
Proteasome subunit beta type-4	PSB4_HUMAN	29	4	27%	2
40S ribosomal protein S4	RS4X_HUMAN	30	4	19%	2
Autophage-related ptotein 101	ATGA1_HUMAN	25	4	19%	2
Tumor protein D52-like 2	Q5JWU6_HUMAN	25	3	17%	2
Microtubule-associated protein RP/EB family member	MARE1_HUMAN	30	3	16%	2
Proteasome subunit alpha type	H0YL69_HUMAN	26	3	17%	2
General transcription factor II	Q86U51_HUMAN	30	3	14%	2
Obg-like ATPase 1	J3KQ32_HUMAN	47	10	25%	2
Phosphoglycerate kinase	B7Z7A9_HUMAN	41	9	71%	2
3-ketoacyl-CoA thidase, mitochondrial	YHIM_HUMAN	42	12	29%	2
Flotillin-1	FLOT1_HUMAN	47	8	49%	2
Sepin H1	SEEPH_HUMAN	46	3	31%	2
Phosphoglycerate mutase 1	PGAM1_HUMAN	29	7	39%	2
Endoplasmic reticulum resident protein 29	ERP29_HUMAN	29	5	24%	2
U2 small nuclear ribonucleoprotein	RU2A_HUMAN	28	3	39%	2
Ras association domain-containing protein	RASF3_HUMAN	28	3	39%	2
Fructose-2, 6-bisphosphatase T1GAR	T1GAR_HUMAN	30	6	27%	2
Galectin-3	LEG3_HUMAN	26	6	29%	2
Isoform 4 of Dehydrogenase/reductase SDR family member	DHRS4_HUMAN	26	9	46%	2
Isoform 2 of V-type protein ATPase subunit E	VATE1_HUMAN	24	3	18%	2
Mitotic checkpoint protein BUB3	B4DDM6_HUMAN	28	3	15%	2
DnaJhololog subunit C member	DNJC8_HUMAN	30	3	14%	2
14-3-3 protein gamma	1433G_HUMAN	28	3	13%	2
Peroxiredoxin-1	PRDX1_HUMAN	22	8	44%	2
Ras-related protein Rap-1A	RAP1A_HUMAN	21	5	32%	2
High mobility group protein B2	HMGB2_HUMAN	24	3	25%	2
Isoform 4 o cellular nucleic acid-binding protein	CNBP_HUMAN	20	3	21%	2
Stromal cell-derived factor 2	SDF2_HUMAN	23	3	22	2
ADP-ribosylation factor-like protein 3	ARL3_HUMAN	20	3	27%	2
Transgelin	TAGL_HUMAN	23	4	20%	2
Very long-chain-specific acyl-CoA dehydrogenase mitochondrial	F5H2A9_HUMAN	73	14	25%	2
Prelamin-A/C	LMNA_HUMAN	74	13	44%	2
Far upstreat element-binding protein 2	FUB2_HUMAN	73	7	14%	2
Far upstream element-binding protein 1	FUBP1_HUMAN	68	7	15%	2
Fibrinogen beta chain	F1BB_HUMAN	56	3	51%	2/2L
Isoform 2 of adenylyl cyclase-associated protein 1	CAP1_HUMAN	52	8	36%	2
Dihydrolopoyl dehydrogenase mitochondrial	DLDH_HUMAM	54	8	25%	2
tRNA-splicing ligase RtcB homolog	RTCB_HUMAN	55	7	35%	2
Elongation factor 1-alpha 1	EF1A1_HUMAN	50	3	26%	2
FAD_AMP lyase	H0YCY6_HUMAN	55	3	8%	2
Isoform 2 of Ig mu chain C region	IGHM_HUMAN	52	3	16%	2
Fascin	FSCN1_HUMAN	55	7	22%	2/2L
Isocitrate dehydrogenase	B4DFL2_HUMAN	45	7	30%	2
Isoform 2 of Testin	TES_HUMAN	47	4	27%	2
Isoform cytoplasmic of glutathione reductase	GSHR_HUMAN	52	4	37%	2
Serpin H1	SERPH_HUMAN	46	3	24%	2
Septin-7	E7EPK1_HUMAN	51	3	8%	2
Fumarylacetoacetase	FAAA_HUMAN	46	13	42%	2
Aspartate aminotransferase, cytoplasmic	AATC_HUMAN	46	12	36%	2
Glutamine synthetase	GLNA_HUMAN	42	6	12%	2
26s protease regulatory subunit 10B	PRS10_HUMAN	44	5	37%	2
Isovalery 1 Coenzyme A dehydrogenase	J3KR54_HUMAN	47	4	26%	2
Medium-chain-specific acyl-CoA dehydrogenase, mitochondrial	B7Z9I1_HUMAN	42	4	42%	2
Isoform 2 of alpha-1-antitrypsin	A1AT_HUMAN	40	4	14%	2
26s protease regulatory subunit 8	A8K323_HUMAN	45	4	15%	2
Isoform 2 of Selenium-binding protein 1	SBP1_HUMAN	45	4	12%	2
Isoform 2 of Putative N-acetylglucosamine-6-phosphate deacetylase	NAGA_HUMAN	47	4	16%	2
Tubulin beta chain	E7EWR1_HUMAN	40	6	33%	2
Alpha-centractin	ACT2_HUMAN	43	3	19%	2
Isoform 2 of Transforming growth factor beta-1-induced transcript 1 protein	TGFI1_HUMAN	48	3	13%	2
Plasmingen activator inhibitor	PAI1_HUMAN	45	3	11%	2
Ig gamma-1 chain c region	1GHG1_HUMAN	36	14	53%	2
Poly(rC)-binding protein 1	PCBP1_HUMAN	37	6	24%	2
Isoform 2 of Arginase-1	ARGI1_HUMAN	36	6	25%	2
ANNEXIN	B4dt77_human	38	5	20%	2
Fructose-bisphosphate aldolase c	ALDOC_HUMAN	39	5	29%	2
Methionine adenosyltransferase 2 subunit beta	MATZB_HUMAN	38	5	21%	2
Isoform 2 of 3-hydroxyisobutyryl-CoA hydrolase, mitochondrial	HIBCH_HUMAN	38	5	16%	2
Cysteine and histidine-rich domain-containing protein	CHRD1_HUMAN	37	4	16%	2
Mitochondrial import receptor subunit TOM40 homolog	TOM40_HUMAN	38	3	18%	2
Proliferation-associated protein 2G4	F8VRZ3_HUMAN	33	3	12%	2
Fructose-bisphosphate aldolase B	ALDOB_HUMAN	39	3	13%	2
Poly-binding-splicing factor PUF60	HOYEM1_HUMAN	36	3	14%	2
Tropomyosin 2 (beta)	Q5TCU3_HUMAN	33	3	11%	2
Electron transfer flavoprotein subunit beta	ETFB_HUMAN	28	12	42%	2
Guanine nucleotide-binding protein subunit beta-2-like 1	GBLP_HUMAN	35	10	50%	2
Coiled-coil-helix-coiled-coil-helix domain-containing protein 3	C9JRZ6_HUMAN	27	9	32%	2
Calcyclin-binding prtein	CYBP_HUMAN	26	9	55%	2
Glyceraldehyde-3-phosphate dehydrogenase	E7EUT4_HUMAN	32	8	43%	2
Isoform 2 of Triosephosphate isomerase	TPIS_HUMAN	27	8	44%	2
Protein NipSnap homolog 3A	NPS3A_HUMAN	28	7	46%	2
Proteasome subunit alpha type-7	PSA7_HUMAN	28	7	42%	2
Serine/arginine-rich-splicing factor 1	J3KTL2_HUMAN	28	7	31%	2
Sepiapterin reductase	SPRE_HUMAN	28	6	32%	2
14-3-3 protein zeta/delta	14332_HUMAN	28	6	32%	2/2L
Adenylate kinase 2, mitochondrial	F8W1A4_HUMAN	26	5	24%	2
Carbonyl reductase [NADPH]	CBR1_HUMAN	30	5	22%	2
Four and a half LIM domain 1	Q5JX18_HUMAN	29	5	25%	2
Heterogeneous nuclear ribonucleoprotein A3	E7EWI9_HUMAN	34	5	19%	2
Ras suppressoe protein 1	RSUI_HUMAN	32	4	19%	2
Heterogeneous nuclear ribonucleoprotein A1	F8VRQ1_HUMAN	33	4	15%	2
Voltage-dependent anion-selective channel protein 3	VDAC3_HUMAN	31	4	23%	2
Isoform 2 of cathepsin S	CAT_HUMAN	32	4	19%	2
Carbonic anhydrase 1	CAH1_HUMAN	29	3	21%	2
3-ketoacyl-CoA thiolase, peroxisomal	H7C131_HUMAN	30	3	22%	2
Biglycan	A6NLG9_HUMAN	35	3	11%	2
Complement C1q subcomponent subunit B	C1QB_HUMAN	27	3	15%	2
Dihydrolipoyl dehydrogenase mitochondrial	DLDH_HUMAN	54	3	7%	2L
Leukocyte elastase inhibitor	ILEU_HUMAN	43	19	33%	2L
Complement factor H-related protein 1	FHR1_HUMAN	38	7	22%	2L
DnaJ hololog subfamily B member 11	DJB11_HUMAN	41	7	24%	2L
Macrophage-capping protein	B4DU58_HUMAN	36	4	13%	2L
Annexin A5	ANXA5_HUMAN	36	2	8%	2L
Isoform short of 14-3-3 protein beta	1433B_HUMAN	28	2	9%	2L

^*^1: from colon cancer tissue of patient 1, male, colon cancer, stage II. 2: from colon cancer tissue of patient 2, female, colon cancer, stage IV; 2L: from liver metastatic tissue of patient 2.

We mapped these proteins using pathway analysis tools [10]. The 170 proteins mapped to a wide array of pathways, e.g., p53, Wnt, PI3K, apoptosis signaling, cadherin signaling, EGF receptor signaling, FGF signaling, FAS signaling, or the RAS pathway. For example, maspin and DAI1 are involved in the p53 pathway; GBB1 is involved in the Wnt and PI3K pathway; 1433G is involved in EGF receptor signaling and FGF signaling pathways; HSP7C, XIAP, FUBP1, RASF3, and PCBP1 are involved in cell apoptosis processes; and HSP7C, PCOC1, CFAD, HPT, FHR1, IN35, C1QB, PRDX1, and CL1C3 are involved in immune responses.

Among the 170 serum-reactive proteins, 4 were reproducibly detected in samples from both cancer patients, including the colon cancer tissue from both patients and the liver metastatic tissue from the patient with liver metastasis. They are maspin (mammary serine protease inhibitor), annexin A3 (ANXA3), leucine aminopeptidase 3 (LAP3), and proteasome subunit alpha type 1 (PSMA1). Because of their reproducibility, these 4 proteins were investigated further in greater depth.

### Validation of maspin, LAP3, ANXA3, and PSMA1 expression by Western blotting

**Figure 3:**
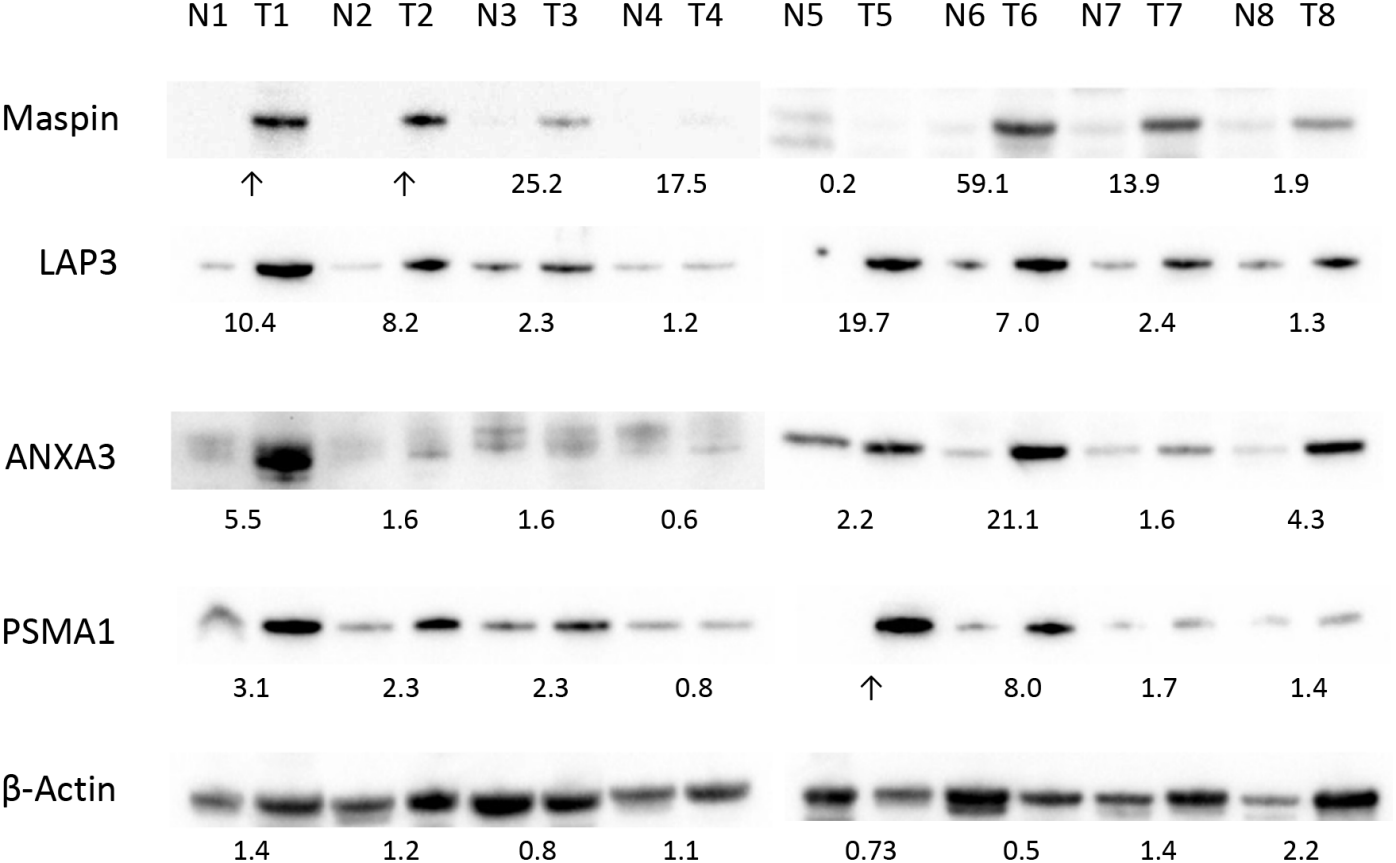
Differential expression of maspin, ANXA3, LAP3 and PSMA1 in colon cancer tissue validated by western blotting Protein extracts from 8 pairs of colon tumor (T1-8) and normal tissue (N1-8) were separated by SDS PAGE and blotted with commercial antibodies specific to each protein. Expression of β-actin in each tissue protein extract was used as a control. The numbers under each pair of blots represent the relative expression ratios, with ratios <1, =1, >1 indicating decreased, unchanged, or increased expression in tumor cancer, respectively.

We first examined the differential expression levels of maspin, ANXA3, LAP3, and PSMA1 in normal colonic mucosa vs. colon adenocarcinoma by 1D Western blotting (Figure 3). Fresh tissues were obtained from a validation set of 8 patients (Table 2). Colonic adenocarcinoma tissue and adjacent normal mucosa were dissected, and proteins were extracted from both. Protein extracts were separated in 2D gels and transferred to PVDF membranes. Protein expression of maspin, LAP3, ANXA3, and PSMA1 was detected with antibodies specific against each individual protein. As shown in Figure 3, the bands detected by Western blotting were at 42 kDa for maspin, 56 kDa for LAP3, 36 kDa for ANXA3, and 30 kDa for PSMA1. The β-actin-normalized relative protein expression ratio ER>1, =1, or <1 represents increased, unchanged, or decreased expression in cancer relative to matched benign mucosa, respectively.

**Table 2:** Patient clinical data for specimens used in the study

Patient	Sample	Age	Gender	Diagnosis	Tumor stage (pTNM)
1	Colon	72	M	Adenocarcinoma	II (pT3 pN0)
2	Colon, liver metastasis	46	F	Adenocarcinoma	IV (pT3 pN2b pM1a)
3	Colon, lung metastasis	60	M	Adenocarcinoma	IV (pT3 pN1a pM1a)
4	Colon	27	F	Adenocarcinoma	I (pT2 pN0)
5	Colon, liver metastasis	43	F	Adenocarcinoma	IV (pT3 pN2a pM1b)
6	Colon	62	F	Adenocarcinoma	III (pT3 pN1c)
7	Colon	69	F	Adenocarcinoma	III (ypT3 ypN2a)
8	Colon, liver metastasis	70	M	Adenocarcinoma	IV (pT3 pN1b pM1a)

Overall, expression of maspin, ANXA3, LAP3, and PSMA1 was increased in colon cancer tissue extracts. In 7 of 8 cases, maspin displayed much higher levels of protein expression in colon cancer (Figure 3, top row). LAP3 expression was higher in 8 of 8 colon tumor tissues than in matched normal tissue, with mean ER of 6.6 (Figure 3, second row). For ANXA3, 7 of 8 patients displayed higher protein expression in cancer, with mean ER of 4.8 across all 8 pairs (Figure 3, third row). For PSMA1, 7 of 8 patients showed overexpression in cancer (Figure 3, fourth row).

### Validation of maspin, LAP3, ANXA3, and PSMA1 expression by IHC

The initial proteomic experiments and the Western blot follow-up in an independent cohort both indicated differential expression of these 4 proteins between normal and cancer. Next, we investigated how these potential markers are distributed at tissue and cellular levels. To this end, we used immunohistochemistry to examine maspin, ANXA3, LAP3, and PSMA1 protein tissue distribution in these 8 patients.

**Figure 4:**
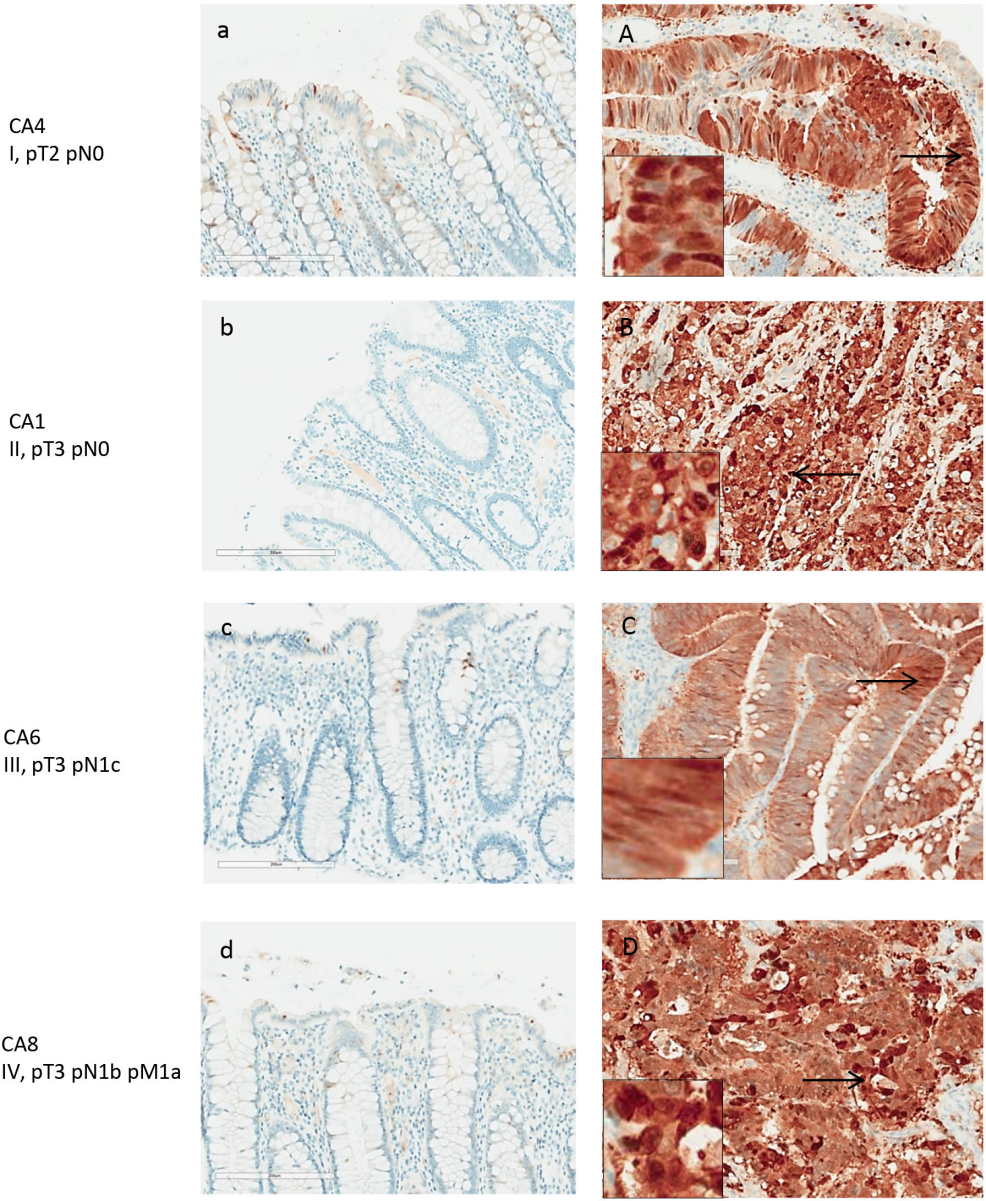
Maspin expression in normal and adenocarcinoma colon tissue by IHC Maspin is stained with anti-maspin (brown), and cell nuclei were counterstained with hematoxylin (blue). Maspin expression is correlated with intensity of the brown stain. Paired tissue samples from 4 patients with stage I-IV colon cancers are shown. Left panels **(a-d)** are the normal colon tissue in each pair, and right panels **(A-D)** are the matched colon tumor tissue. Maspin expression is significantly increased in the cancer tissue in each patient.

Maspin expression was significantly increased in primary colon carcinoma but barely detectable in matched normal colonic mucosa (Figure 4A-4D). 5 colon cancers showed strong expression of maspin and 3 showed medium level expression. In contrast, in the 8 matched normal mucosal tissues, maspin expression was negative in 6 and weak in 2. As assessed by semi-quantitative scoring of the immunostaining, the average score for maspin expression was 2.6 in colon cancer tissues and 0.1 in normal colon tissues (Figure 8).

**Figure 5:**
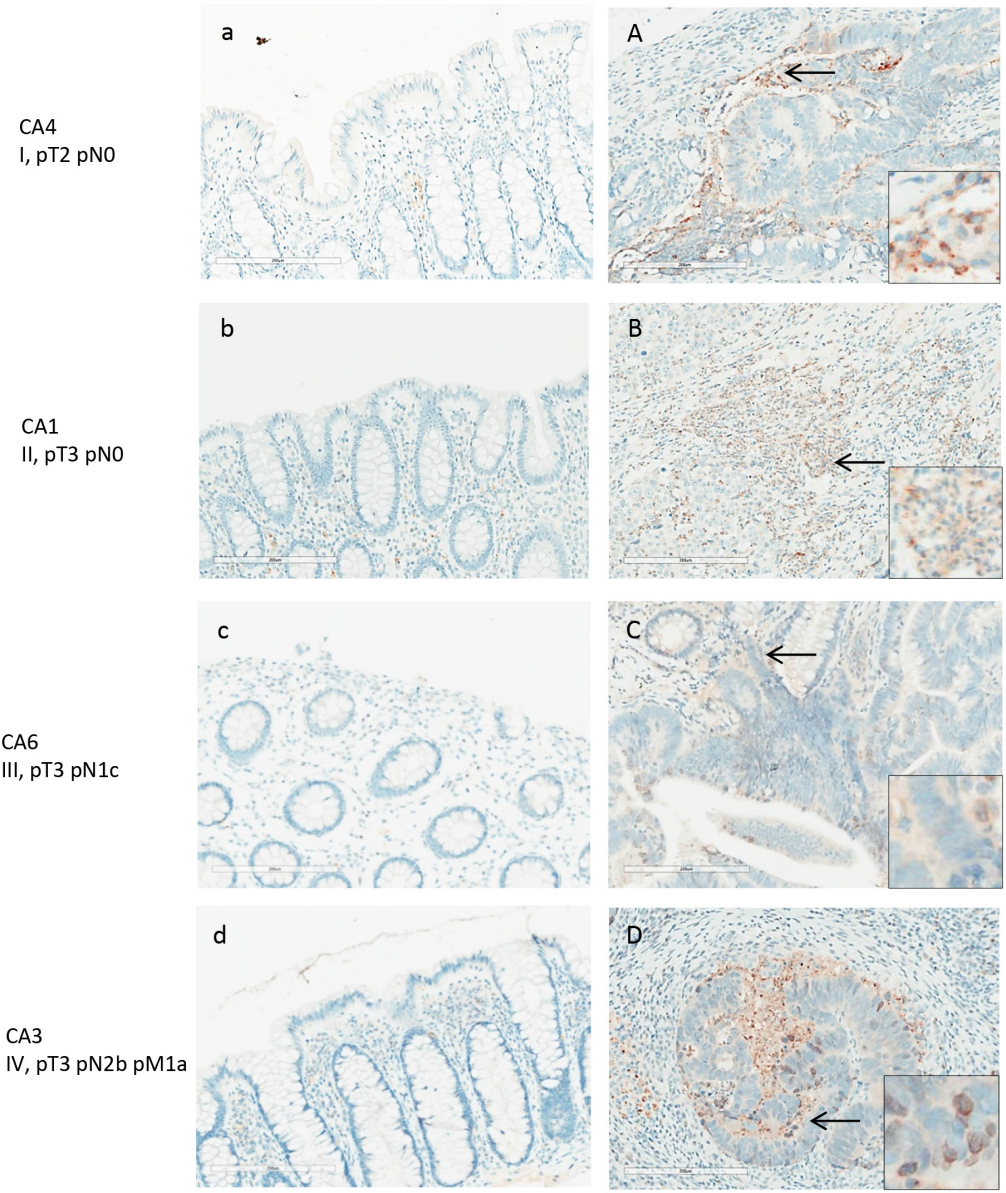
ANXA3 expression in normal and adenocarcinoma colon tissue by IHC Colon cancer tissues **(A-D)** and adjacent normal tissues **(a-d)** shown are from patients with stage I-V colon adenocarcinomas. Weak to moderate expression of ANXA3 was detected in colon cancer cells and the stroma. ANXA3 appeared primarily in the cytoplasm of cancer cells (inserts in A, D).

**Figure 6:**
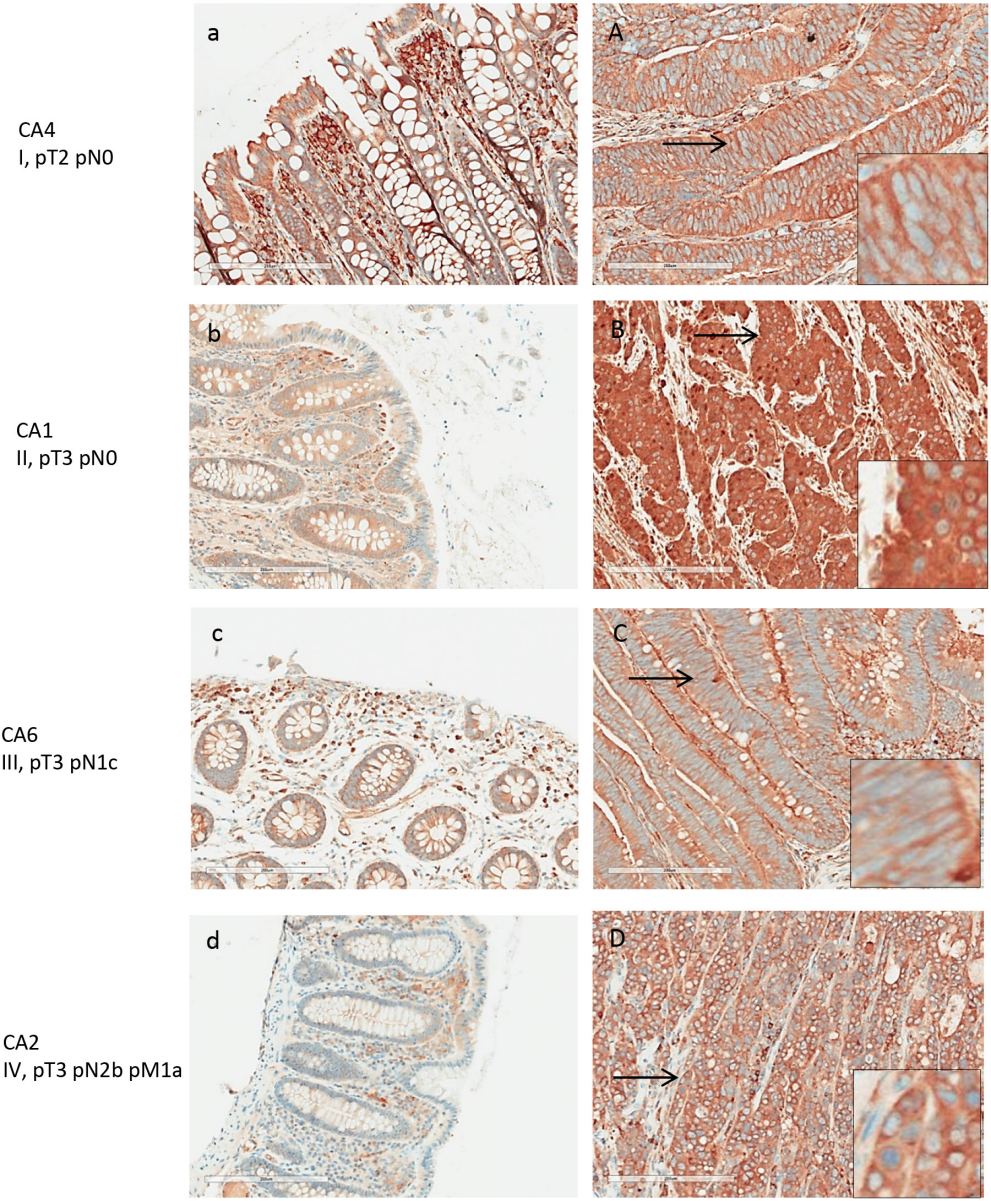
LAP3 expression in normal and adenocarcinoma colon tissue by IHC Tissue from patients with stage I-V colon cancer were examined. Left panels **(a-d)** are normal colonic tissues, and right panels **(A-D)** are the matched colon cancers. LAP3 expression was detected in lymphocytes of the normal colon tissue at moderate intensity. Strong expression was detected in colon cancer cells, primarily in the cytoplasm.

**Figure 7:**
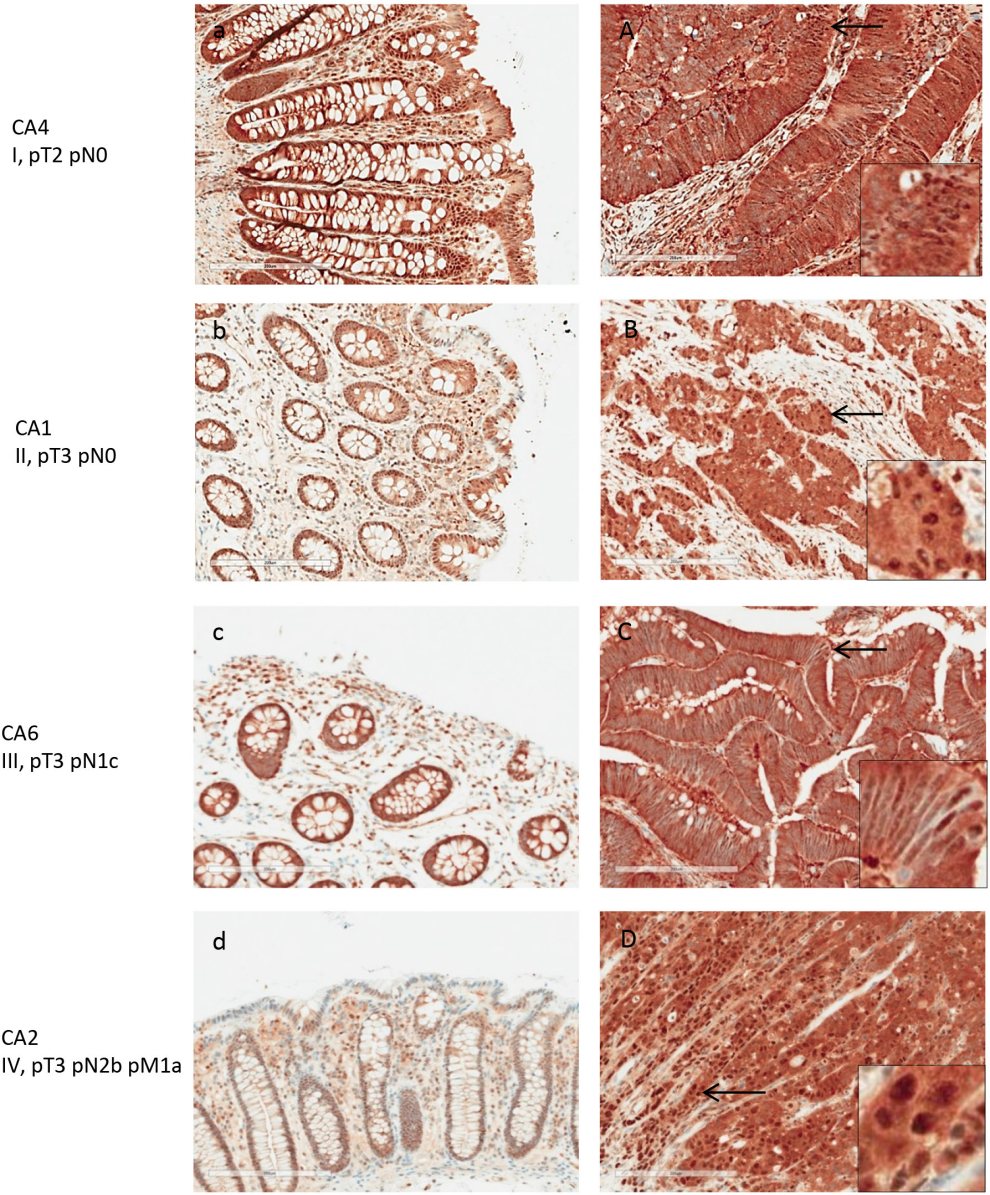
PSMA1 expression in normal and adenocarcinoma colon tissue by IHC Matched pairs of normal **(a-d)** and tumor **(A-D)** tissues from patients with stage I-V colon cancers are shown. PSA1 expression was present in normal colon tissue at moderate levels but increased significantly in cancer tissue.

**Figure 8:**
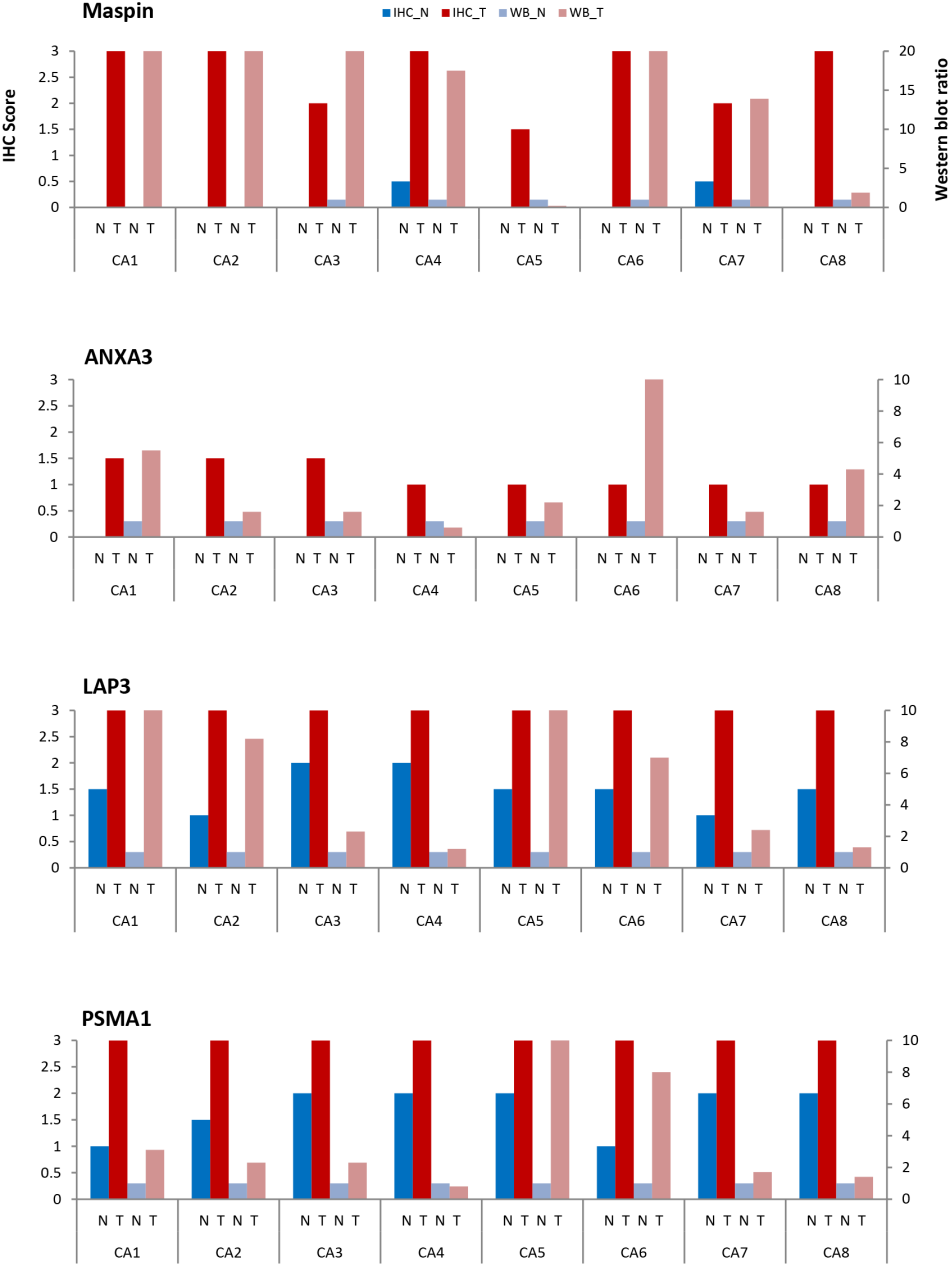
Comparison of maspin, ANXA3, LAP3, and PSMA1 expressions by IHC and Western blotting in 8 patients Normal (N) and tumor (T) colon tissue pairs from 8 patients (CA1-CA8) are examined. Paired tissue were examined by IHC (normal tissue – dark blue; tumor tissue – dark red). Protein extracts were examined by Western blotting (normal – light blue; tumor – light red). Expressions were scored according to the average staining intensity on a 0-3 scale for IHC (left Y axis) and a 0-10 expression ratio scale for Western blotting (right Y axis), with normal tissue scaled to 1.

Maspin protein was mainly found expressed in the neoplastic colon cancer cells (Figure 4A-4D), both in the cytoplasm and nuclei. Poorly differentiated cancer cells showed stronger nuclear staining of maspin than well or moderately differentiated cancer cells. Strongly expressed nuclear maspin was also detectable in tumor cells at the tumor’s invasive front. Maspin staining was generally negative in normal colonic mucosa, with few epithelial cells showing weak staining (Figure 4A-4D). Overexpression of maspin was evident in an early stage I patient (Figure 4A), suggesting that maspin overexpression can occur early.

ANXA3 was detected in the colon cancer tissues of all 8 patients, although the overall expression levels were of weak to medium intensities and predominantly cytoplasmic (Figure 5A-5D). In all 8 normal mucosal tissues, ANXA3 expression was absent or barely detectable. Although its expression in cancer tissue was not particularly strong, ANXA3 did increase an average 1.2-fold in cancer tissue relative to the normal counterpart as assessed by semi-quantitative assessment (Figure 8). The ANXA3 expression increase appeared to remain at similar levels with no further increase in advanced stages (Figure 5C-5D).

LAP3 expression was present in both normal and colon cancer tissue (Figure 6). Among the 8 patients tested, all colon cancer tissue displayed strong expression of LAP3. LAP3 was present in colon cancer cells as well as the surrounding stroma. LAP3 was also found in lymphocyte infiltrates. LAP3 expression was visibly increased in colon cancer tissue (Figure 6A-6D) when compared with normal colonic mucosa. Semi-quantitative assessment of LAP3 expression revealed an average score of 3 for colon cancer tissues and 1.5 for normal colonic mucosa.

PSMA1 expression was markedly increased in colon cancer tissue, although it was also detectable in normal colonic mucosa (Figure 7). All 8 colon cancer tissue showed strong immunoreactivity with PSMA1. Both nuclear and cytoplasm showed strong labeling. Semi-quantitative assessment of PSMA1 expression yielded an average score of 3 for colon cancer and 1.7 for normal colonic mucosa (Figure 8).

## DISCUSSION

In this study, we focused on discovering abnormally immunogenic proteins in colon cancer that induced circulating antibodies in the patient’s own serum. We were motivated by the hypothesis that cancer-specific immunogenic molecules are promising markers for cancer diagnosis as well as immunotherapeutic development. Cancer development is dictated by progressive changes in the molecular makeup of cancer cells and the surrounding tissue milieu. While most of the molecular changes may be subtle deviations from the norm and regarded as harmless by the immune surveillance, cancer-specific changes that trigger a humoral immune responses may hold significant clues towards understanding the intricate interactions between cancer and the immune defense.

We developed an immuno-proteomic strategy by combining 2D serum antibody-capture with state-of-the-art proteomics methods. Using a discovery set, about 80 protein spots were found to be expressed differentially between normal colonic mucosa and cancer. Upon sequencing these spots by mass spectrometry, 170 proteins were identified in tumor tissue only but were not found in matched normal mucosa (Table 1). Of these, maspin, ANXA3, LAP3, and PSMA1 were reproducibly discovered as serum-reactive in all 3 initially tested cancer samples, including colon tumor tissue from the 2 patients and the liver metastatic tissue from the patient with primary colon cancer and secondary liver metastasis. These proteins were hence examined in greater detail. Overexpression of these 4 proteins in cancer relative to normal was then independently confirmed by Western blotting and immunohistochemistry in a validation set of 8 patients spanning various clinical stages of colon cancer.

To our knowledge, this study is the first to report PSMA1 overexpression in colorectal cancer. PSMA1 has been reported to be overexpressed in breast cancer [17], and PSMA1 gene expression levels have been reported to be upregulated in a neuroendocrine pulmonary tumor animal model [18]. PSMA1 (proteasome subunit alpha 1) is one of the 17 essential subunits of the 20S proteasome, a multi-catalytic proteinase complex with a highly ordered ring-shaped core structure. Another member of the proteasome, PSB7 (proteasome subunit beta 7), has previously been reported by our group to be up-regulated in colorectal cancer as a potential mechanism for immune escape [11].

To our knowledge, our present study is also the first to report LAP3 overexpression in colorectal cancer. LAP3 (leucine aminopeptidase 3) belongs to the aminopeptidase family that comprises enzymes that catalyze the hydrolysis of leucine residues at the N-terminus of peptides and proteins [19]. LAP3 is presumably involved in the processing and regular turnover of intracellular proteins. Our Western blot and IHC results showed that LAP3 protein was much more abundant in the cancerous cells compared to matched normal colonic mucosa. Dysregulation of LAP3 expression could alter peptide activation, thus leading to changes of cell proliferation, invasion, and angiogenesis [20, 21]. Studies have shown that LAP3 is overexpressed in several malignant tumors, including ovarian epithelial malignancy, gliomas, esophageal squamous cell carcinoma, and hepatocellular carcinoma [21–24].

ANXA3 expression has been previously observed to correlate significantly with tumor growth and poor prognosis in colorectal cancer [25, 26]. However, cancer autoantigenic properties of ANXA3 have not previously been described. ANXA3 is a member of the annexin family, which binds membrane phospholipids in a calcium-dependent manner and plays a role in the regulation of cellular growth and in signal transduction pathways. ANXA3 participates in multiple biological activities, including cell apoptosis, cell proliferation, cell differentiation, signal transduction, anti-inflammatory response, endocytosis and exocytosis, which may contribute to cancer development and metastasis [25, 27]. Upregulation of ANXA3 was also found in prostate, lung, gastric, and hepatocellular cancers [27, 28]. ANXA3 expression was strongly associated with tumor size, stage, and poor prognosis [27]. ANXA3 correlates also with cell proliferation as indicated by Ki-67 and Bcl-2 expression [27] and appears to be preferentially expressed in cancer stem-like cells/cancer initiating cells (CSCs/CICs) [28].

Maspin as a protein marker for colorectal cancer has been examined by several studies [29–32]. However, maspin’s cancer immunogenic property, as described in our study, has not previously been reported. Maspin expression has also been observed to be upregulated in pancreatic, gallbladder, and thyroid cancers [33–35] but down-regulated in breast, prostate, and gastric cancers and melanomas [33–35]. Maspin was identified as a marker of serrated colorectal polyps [36]. Nuclear maspin expression was reported to correlate with tumor aggressiveness in colorectal cancer [37] and may be a predictive marker for fluorouracil treatment response in colon cancer [38]. Maspin was also reported as a CEA-interacting marker for colon cancer [39] and a marker for early recurrence in stage III and IV colon cancers [40]. Some studies claimed that subcellular localization of maspin expression in cytoplasm or nucleus correlates with colon cancer aggressiveness and treatment outcome [41, 42]. A large clinical study however reported that maspin expression was correlated with few other conventional histopathology variables and was not a significant prognostic factor in advanced stage disease [43]. Overall, the role of abundance of maspin expression in colon cancer remains currently not well defined. In so far, our discovery of a serum immunogenic property of maspin may point to another parameter that can be examined beyond simple abundance changes of the protein in the tumor.

## MATERIALS AND METHODS

### Clinical tissue and serum collection

All specimens used in this study were collected by the BioSpecimen Science Program at the University Health Network (UHN), Toronto, ON, Canada, according to institutional consent and IRB protocols. Samples were obtained from patients diagnosed with various stages of colonic adenocarcinoma (Table 2). Matched sets of specimens were collected from each patient, including primary carcinoma tissue and adjacent normal colonic mucosa, metastatic tissue (where applicable), and serum. Fresh tissues were dissected immediately after surgical removal from patients, flash frozen in liquid nitrogen, and stored at −80°C. Portions of tissues were also fixed in formalin and embedded in paraffin. Serum samples were stored at 4°C. Samples from patients 1 and 2 were used in the initial discovery studies, and further validation studies were conducted with samples from all 8 patients.

### Protein extraction

Aliquots of ∼200 mg of tissue were suspended in 1 mL of a protein extraction solution containing 0.15 M NaCl, 50 mM Tris-HCl (pH 7.4), 1.0% NP-40, and Complete Mini Protease Inhibitor Cocktail (Roche Diagnostics). Tissue samples were mechanically homogenized on ice and then sonicated with 10 sec on/10 sec off for 10 cycles at 4°C. The homogenates were centrifuged at 14,000 rpm in a bench top centrifuge at 4°C for 60 min. The supernatants were filtered through 0.45-μm syringe filters. The filtrates containing total soluble proteins were used for 1D and 2D gel electrophoresis analyses. Protein concentrations were measured with the Pierce BCA Protein Assay Kit (Thermo Fisher Scientific) with bovine serum albumin (BSA) as the standard. All extracted protein samples were stored at −80°C until further analysis.

### 1D and 2D gel electrophoresis

One dimensional (1D) SDS-PAGE analysis was performed with a SureLock Xcell system (Thermo Fisher Scientific). Protein samples were mixed with 25% NuPAGE Sample Buffer and 10% Reducing Agent and heated at 70°C for 10 min. About 20 μg of proteins were loaded into wells of 4%-12% acrylamide NuPAGE Bis-Tris gels. The proteins were separated at 200 V for 35 mins in MES SDS running buffer. Gels were stained with Bio-Safe Coomassie Blue G250 and scanned in a ChemDoc MP imaging system (Bio-Rad).

For 2D gel electrophoresis, 200-μg samples of proteins were first cleaned with the ReadyPrep 2D Cleanup Kit (Bio-Rad) or by precipitation with acetone and then dissolved in 185 μL of ReadyPrep rehydration buffer. Proteins were separated first by isoelectric focusing in 11-cm IPG (immobilized pH gradient) strips (pH 3 to 10; Bio-Rad). Proteins were then separated in the second dimension by SDS PAGE with 8-16% gradient Criterion Tris-glycine polyacrylamide gels and Tris-glycine SDS buffer (Bio-Rad). Gels were stained with Bio-Safe Coomassie Blue G250 and scanned in a ChemDoc MP imaging system.

### Protein sequencing by LC-MS

Protein spots of interest were manually excised from 2D gels and fragmented by in-gel tryptic digestion. In brief, the gel spots were washed with 50 μL of 50 mM NH_4_HCO_3_ (pH 8.0) and treated with 50 μL of 25 mM NH_4_HCO_3_ and 50% acetonitrile for 10 minutes. They were reduced with 10 mM dithiothreitol for 30 min and alkylated with 100 mM iodoacetamide for 15 min. The gel spots were then incubated with 13 μg/L trypsin at 37°C for 3 h. The digested peptides were extracted from the gel with 50 μl of 50% acetonitrile and 5% formic acid, dried in a Speed-Vac, and dissolved in 5 μL of 0.1% formic acid.

The peptides were loaded into a 150-μm ID Magic C_18_ pre-column at 4 μL/min and separated in a 75-μm Magic C_18_ analytical column (Michrom Biosciences, Auburn, CA). Peptides were eluted with a gradient of 0-40% acetonitrile in 0.1% formic acid at 0.3 μL/min over 60 min through an EASY n-LC nano-chromatography pump (Proxeon Biosystems). Eluted peptides were analyzed in an LTQ-Orbitrap hybrid ion trap-orbitrap mass spectrometer (Thermo Scientific) operated in a data-dependent acquisition mode [8]. MS was acquired at 60,000 FWHM resolution in the Fourier transform MS. MS/MS was carried out in the linear ion trap, and 6 MS/MS scans were obtained per MS cycle.

All MS/MS data were analyzed with Mascot (Matrix Sciences), Sequest (Thermo Scientific), and X!Tandem (The GPM) using the Human UniProt database with digestion enzyme set to trypsin. Searches were carried out with a fragment ion mass tolerance of 0.60 Da and a parent ion tolerance of 10.0 ppm. Scaffold was used to validate MS/MS-based peptide and protein identifications. To ensure the highest quality of protein identification, only peptide identifications that exceeded 95% thresholds were accepted. Protein identifications were accepted if they contained more than two different identified peptides. Bioinformatics analyses and protein function searches were conducted using the UniProtKB database and NCBI BLAST [16].

### Western blotting

Proteins from 1D or 2D gels were transferred to PVDF membranes with Midi Transfer Packs in a Trans-Blot Turbo Transfer System (Bio-Rad). The membranes were blocked with 5% (m/v) BSA in phosphate buffed saline (PBS, pH 7.4) and 0.5% (v/v) Tween 20 at room temperature for 2 h, and then incubated with primary antibodies at room temperature for 2 h or at 4°C overnight. The primary antibodies were used at the following dilutions: patients’ sera at 1:1,000, anti-maspin (clone G167-70, 554292; BD Pharmingen) at 1:500, anti-ANXA3 (sc-101885, Santa Cruz Biotechnology) at 1:500, anti-LAP3 (ab154809, Abcam) at 1:1,000, anti-PSA1 (ab109500, Abcam) at 1:1,000, and anti-β-actin at 1:10,000 dilution. The membranes were then incubated with secondary antibodies at room temperature for 1 h. The secondary antibodies used include goat anti-human IgG-HRP (horseradish peroxidase) (sc-2453, Santa Cruz Biotechnology) at 1:3,000 dilution, goat anti-mouse IgG-HRP (sc-2005) at 1:10,000 dilution, and goat anti-rabbit IgG-HRP (sc-2004) at 1:4,000 dilution. In between all steps, membranes were washed 3 times with PBS and 0.5% Tween 20. Membranes were developed with Clarity Western ECL Substrate (Bio-Rad). Membrane images were captured in a ChemiDoc MP imaging system (Bio-Rad).

The intensities of protein bands was analyzed with ImageJ software. The density (D) of a band was measured as Dpc (protein from cancer tissue) or Dpn (protein from normal tissue). The protein expression ratio between cancer and normal samples was calculated as the relative density RDp=Dpc/Dpn. The protein expression ratio (ER) was then calculated by normalizing the respective protein’s ratio (RDp) with respect to the β-actin expression ratio (RDa) as ER=RDp/RDa [11].

### Immunohistochemistry (IHC)

Paraffin sections of 4 μm thickness were heated at 60°C overnight before staining. The slides were washed with TBS (pH 7.4), and antigens were retrieved by heating at 95°C for 8 min and boiling at 100°C for 1.5 h in Tris/borate/EDTA buffer (pH 8-8.5). Tissue sections were incubated at 37°C for 1 h with anti-maspin (1:400 dilution), anti-ANXA3 (1:100 dilution), anti-LAP3 (1:400 dilution), and anti-PSMA1 (1:400 dilution) in TBS containing 1% BSA. Antibody reaction was detected with the UltraView Universal DAB Detection Kit (Ventana). The kit contains a cocktail of enzyme-labeled secondary antibodies that locate the bound primary antibody. The complex is then visualized with hydrogen peroxide substrate and 3, 3’-diaminobenzidine tetrahydrochloride (DAB) chromogen, which produces a dark brown precipitate that is readily detected by light microscopy. Cell nuclei were counterstained with Hematoxylin II and Bluing Reagent (Ventana). Immunohistochemical staining was scored according to the average stain intensity: 3 for dark brown color, 2 for medium brown, 1 for weak brown, and 0 for no staining.

## CONCLUSIONS

In summary, this paper reports several new findings. We first developed an innovative immuno-proteomic method that uses antibodies in cancer patient sera to discover cancer-specific immunogens as cancer markers. We then used this approach to discover a set of cancer-immunogenic proteins in colon cancer (maspin, ANXA3, LAP3, and PSMA1) and showed that these proteins are overexpressed in the tumor, pointing to a possible abundance mechanism by which these proteins become immunogenic. Further research using larger cohorts spanning from early to late stages of disease as well as different treatment histories and outcomes will be needed to investigate whether the four proteins are correlated in overexpression, whether they contribute directly or indirectly to the pathobiology of the disease, and whether there are differences between primary and metastatic sites.

We hypothesize that tissue overexpression leads to immunologically abnormally visible proteins and thus induces specific serum antibodies against these abnormalities in the cancer patient. More generally, the totality of neoantigenic proteins in cancer (i.e., the “cancer antigen-ome”) may be a promising avenue not only towards novel diagnostic serologic tests but also the basis for new targets of immunotherapeutic development. Our mass spectrometry-based immune-proteomic method of serum reactivity screening as developed in this paper should be a powerful tool for comprehensively characterizing such cancer autoantigen-omes.

## Abbreviations

ANXA3: annexin A3
IHC: immunohistochemistry
LAP3: leucine aminopeptidase 3
MS: mass spectrometry
PSMA1: proteasome subunit alpha type-1
